# Vulnerability Analysis and Security Assessment of Secure Keyboard Software to Prevent PS/2 Interface Keyboard Sniffing

**DOI:** 10.3390/s23073501

**Published:** 2023-03-27

**Authors:** Kyungroul Lee, Kangbin Yim

**Affiliations:** 1 Department of Information Security Engineering, Mokpo National University, Muan 58554, Republic of Korea; carpedm@mnu.ac.kr; 2 Department of Information Security Engineering, Soonchunhyang University, Asan 31538, Republic of Korea

**Keywords:** secure keyboard software, keyboard security, security assessment, keyboard sniffing, PS/2 interface keyboard, vulnerability analysis

## Abstract

Online security threats have arisen through Internet banking hacking cases, and highly sensitive user information such as the ID, password, account number, and account password that is used for online payments has become vulnerable. Many security companies have therefore researched protection methods regarding keyboard-entered data for the introduction of defense techniques. Recently, keyboard security issues have arisen due to the production of new malicious codes by attackers who have combined the existing attack techniques with new attack techniques; however, a keyboard security assessment is insufficient here. The research motivation is to serve more secure user authentication methods by evaluating the security of information input from the keyboard device for the user authentication, including Internet banking service. If the authentication information input from the keyboard device is exposed during user authentication, attackers can attempt to illegal login or, worst, steal the victim’s money. Accordingly, in this paper, the existing and the new keyboard-attack techniques that are known are surveyed, and the results are used as the basis for the implementation of sample malicious codes to verify both a security analysis and an assessment of secure keyboard software. As a result of the experiment, if the resend command utilization attack technique is used, 7 out of 10 companies’ products expose keyboard information, and only 1 company’s products detect it. The fundamental reason for these vulnerabilities is that the hardware chip related to the PS/2 interface keyboard does not provide security facilities. Therefore, since keyboard data exposure does not be prevented only by software, it is required to develop a hardware chip that provides security facilities.

## 1. Introduction

The damage that is incurred by the Internet banking and telebanking sectors due to eavesdropping is rapidly increasing in the current time period, leading the Ministry of Information and Communication in the Republic of Korea, in collaboration with the Republic of Korea’s Financial Supervisory Commission, Financial Supervisory Service, Ministry of Commerce, and Korea Information Security Agency, to announce “comprehensive countermeasures for strengthening the safety of e-commerce”; moreover, the scales of the financial transactions and eCommerce are increasing [1], and these are becoming parts of the national economy whereby the expectation is that they will become the “blue chips” of I.T. technology. Nevertheless, Internet banking hacking cases occurred during May 2005 in Republic of Korea while eCommerce and online-banking systems were widely used; furthermore, and beginning with this case, the number of online-banking hacking cases has increased up until the time of this paper [2,3,4].

eCommerce security consists of the following three parts: anti-hacking software, a method of electronic-finance operation and management, and a certificate-management system. While the main purpose of the anti-hacking software is the collection and analysis of malicious codes in the eCommerce environment, it is difficult to counteract attacks efficiently because the countermeasures are being performed by individual security companies; furthermore, the execution of security software on the Web browser or extra software in the user mode limits the capability regarding the detection or blocking of malicious codes. For the delivery of secure software, the focus is therefore the protection of the keyboard-input data from the user.

In spite of these security efforts, numerous drawbacks affect the present keyboard-security measures [5,6,7,8,9,10,11] and critical problems are not consistently detected for both the PS/2 keyboard and the USB keyboard [5,6,8,10,11]. Additionally, the present keyboard-security assessment is inadequate, and for this reason, the attack techniques for the PS/2 keyboard are surveyed in this paper, whereby the secure keyboard software that are targeted by the surveyed attack techniques are used as the basis for the verification of the PS/2-keyboard security.

The state-of-the-art keyboard attacks has been researched. In [12], the potential security risks of stealing user’s sensitive keystrokes in Android apps were demonstrated. The authors verified that keystrokes are stolen from popular websites by implementing a proof-of-concept tool. As a result, existing keyboard applications were misused as malicious keyloggers. However, this attack technique steals keyboard data by exploiting Android apps, not a physical keyboard attack technique. Ref. [13] demonstrated that inference attack on physical keyboards using acoustic signals is possible on smartwatch, and [14] presents a keylogging inference attack typed with in-air tapping keyboard. These two studies do not install malicious codes to steal keyboard data without infecting target computers. However, since the acoustic signals are analog signals, these attack techniques are sensitive to data changes caused by noise. Ref. [15] introduced a video-based keystroke inference attack. Nevertheless, this attack technique has a limitation in that a camera for capturing keyboard video is required. Ref. [16] introduced AI-driven thermal attacks on commonly used computer keyboards. However, this attack technique has a limitation in that it requires thermal cameras. In [17], a remote keylogging attack on search engine autocomplete was demonstrated. In [18], vulnerabilities of keystroke sniffing, keystroke injection, forced device pairing, malicious macro programming, and denial of service in 2.4 GHz Wireless Mice and Keyboards were analyzed. In [19], a virtual keystrokes recognition method using channel state information of WiFI signals in VR (Virtual Reality) in VR headsets was introduced. In [20], a keystroke inference attack on an MR (Mixed Reality) device and [21] demonstrated a keylogging inference attack on air-tapping keyboards in virtual environments was introduced. At present, users use a laptop connected with PS/2 interface keyboard than a desktop. Therefore, this article aims to evaluate the security of keyboard data for PS/2 interface keyboard. 

PS/2 keyboard attack techniques include WinProc replacement, message hooking, filter driver insertion, interrupt object replacement, IDT replacement, direct polling, C/D bit vulnerability, utilization, debug exception handler replacement, and RESEND command utilization techniques. Interrupt object replacement technique is the replacement of the interrupt routine by an attacker with a malicious routine to steal the keyboard scan code. Direct polling technique steals keyboard data by periodically reading the keyboard input data from the data port [9]. The C/D bit vulnerability technique is a method of stealing real keyboard data by classifying fake keyboard data revealed due to C/D bit [8]. Ref. [5] proposed a method to solve the C/D bit vulnerability attack technique. This solution has been verified to effectively generate fake keyboard data even if the C/D bit does not make polling edge. Nevertheless, this method exposes keyboard data by RESEND control code utilization attack technique. Debug exception handler replacement technique is a method of stealing keyboard data by neutralizing the debug exception handler that monitors processes accessing the keyboard data port. RESEND control code utilization technique is a method of stealing received keyboard data by directly transmitting the RESEND command to the keyboard [6].

Regarding the USB interface keyboard attack, since the HCCA and the transaction buffers can be read by any software due to the pHCCA register, USB keyboard data is exposed simply by reading the transaction buffers [7]. To prevent the exposure of USB interface keyboard data, a method of deleting the keyboard data stored in the transfer descriptor (TD) buffer pointer or inserting fake keyboard data was proposed [7]. Ref. [4] pointed out the problem of keyboard data exposure in USB interface keyboard and PS/2 interface keyboard and proposed a hardware approach to solve the problem. Therefore, the fundamental reason for these vulnerabilities is that the hardware chip related to the PS/2 interface keyboard does not provide security facilities. Therefore, since keyboard data exposure is prevented only by software, it is required to develop a hardware chip that provides security facilities. Moreover, ref. [10] proposed OpenTC’s secure banking prototype; however, there is a limitation that it does not fundamentally prevent keyboard data exposure. 

As a result of demonstrating on real sites with these attack techniques, 70% of the attack success rate was shown by neutralizing the secure keyboard software running on the system to 7 out of 10 sites, and proof-of-concept tools were not detected by anti-virus programs. Consequently, the keyboard data attack tools proposed in this paper not only effectively steal keyboard data, but also demonstrate that it is extremely effective techniques because the attack tools are not detected by anti-virus programs.

The contribution of this paper is as follows.

- By analyzing the transmission of PS/2 keyboard data, we analyzed the vulnerability points associated with PS/2 keyboard data exposure. Moreover, we analyzed the attack techniques associated with those points and verified the most powerful attack techniques.
- Based on the newly discovered attack technique, we verified the exposure of the keyboard data input from the PS/2 keyboard in the real-world Internet banking service. As a result, we proved the exposure of keyboard data in most Internet banking services.
- The attack techniques described in this paper were not detected by various anti-virus programs. This means that an attacker can easily penetrate the victim’s system and steal keyboard data.

This paper is organized as follows. Section 2 describes the keyboard attack techniques, and we assess the security of secure-keyboard software based on the analyzed keyboard attack techniques and derive the results of keyboard security in Section 3. Section 4 the conclusion of the study.

## 2. Attack Techniques of the PS/2 Keyboard

In terms of online-banking services, a secure channel must first be established between a user and a banking server, and the following security requirements are needed [10,22]:

***Confidentiality***: Only authorized users can access the transferring data.

***Integrity***: The transferring data is not subject to falsification by a third party, and through detection, action is taken to provide protection from an attack.

***Mutual authentication***: The communication-related entities must authenticate each other, and they must also verify the authentication results. 

***Non-repudiation***: The communication-related entities must verify their communication results regarding the denial of third-party behaviors.

Regarding online-banking services, the above four security requirements are sufficiently supported, but because insecure keyboards are used to input the data that are used for these services, critical confidentiality vulnerability has been identified. While confidentiality is ensured for the transmissions between a server and a user, the step before the data transferal wherein the user enters the data does not satisfy the data-confidentiality requirement. Specifically, this problem is an exceedingly serious problem outside of the Republic of Korea, as many other countries do not support the countermeasures that protect keyboard data. In the case of security companies in the Republic of Korea, they are prepared for the possibility of a confidentiality contravention, so secure keyboard software has been installed for online-banking services to protect keyboard data. For this paper, the products that are applied for most of the Websites in the Republic of Korea were chosen for the security assessment.

**Figure 1:**
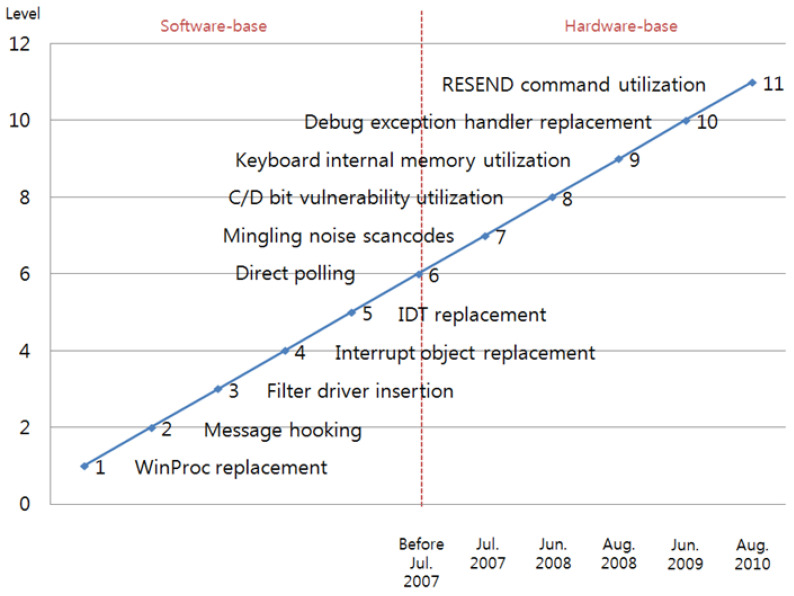
Keyboard attack and protection techniques.

Although secure keyboard software is currently used, attack techniques such as those that are used for the stealing of keyboard data have not been verified, so a keyboard-security assessment is urgently needed. In this paper, the attack techniques for the extortion of keyboard data are consequently surveyed, and the security of the secure-keyboard software is verified. Figure 1 shows the attack techniques that are used for the extortion of keyboard data.

The most popular attack techniques that have resulted in serious vulnerabilities are interrupt-object replacement, direct polling, C/D-bit-vulnerability utilization, debug-exception-handler replacement, and RESEND-command utilization.

### 2.1. Interrupt-Object Replacement

**Figure 2:**
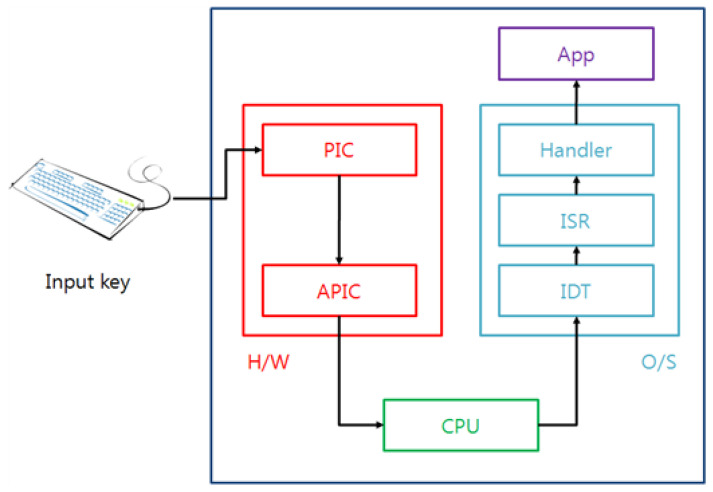
Transferal process of the keyboard data.

Figure 2 shows the transferal process of the keyboard data that are input from the keyboard to the user-mode application software.

**Figure 3:**
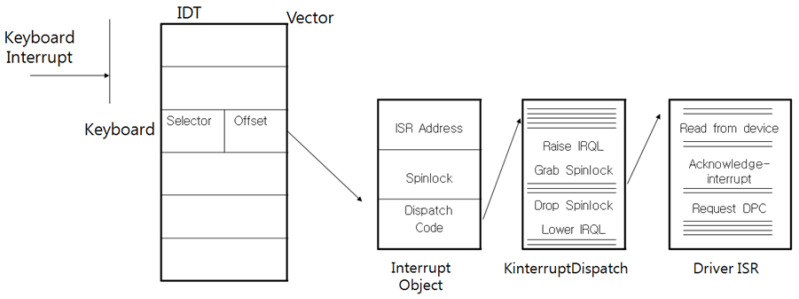
The calling process of the interrupt-service routine of the keyboard in the operating system.

The PIC (Programmable Interrupt Controller) and the APIC (Advanced Programmable Interrupt Controller) are two of the interrupt controllers that are used for the handling of interrupts so that a keyboard interrupt also transfers the keyboard data through the PIC and APIC controllers, because the keyboard is handled by the interrupt. When the keyboard data are input from the keyboard, the PIC identifies the origin of the interrupt, it processes a number of operations to support the O/S (operating system), and then the controller notifies the APIC of the interrupt. The APIC receives the cause of the interrupt and then generates the interrupt to the CPU. In the past, the CPU would execute the whole input and the output operations, but now the CPU prepares extra tables and handlers to perform the required input and output operations for the improvement, effectiveness, and efficiency of the performance; for this reason, when interrupts occur, the CPU communicates with the prepared interrupt handlers. These tables and handlers are called the IDT (Interrupt Descriptor Table) and the ISR (Interrupt Service Routine), and these finally handle the transferal of the data including the executed results to the application program. A representative attack at the O/S level is the replacement of the interrupt routine by an attacker with a malicious routine to steal the keyboard scan code. Scan codes are derived from a keyboard for which the general characters have been transformed into the alphabet in the O/S. Figure 3 shows the calling process of the interrupt service routine in the O/S.

**Figure 4:**
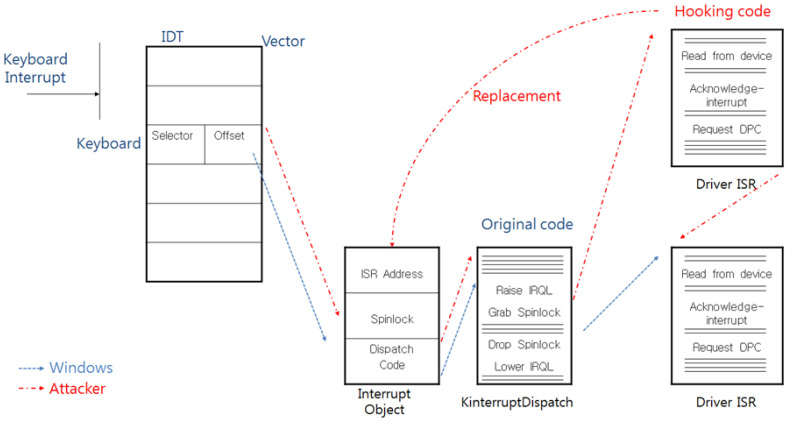
The calling process after the ISR is replaced by the attacker.

**Figure 5:**
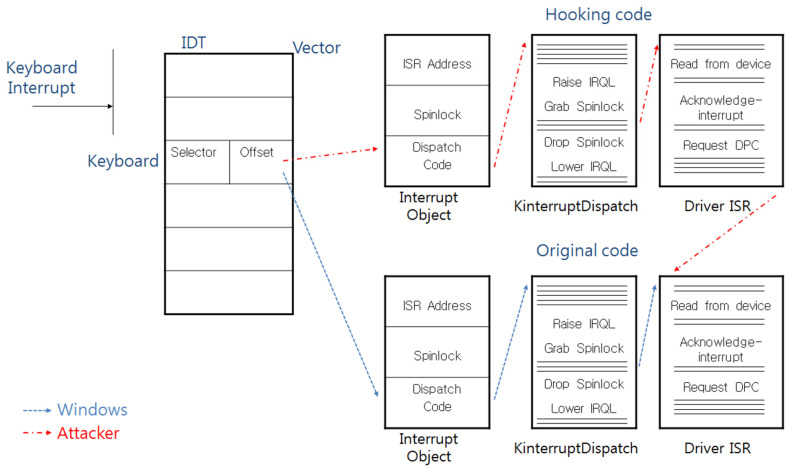
The calling process after the IDT is replaced by the attacker.

As mentioned above, attackers can replace the IDT or the ISR with their code to extort the scan code, and Figure 4 and Figure 5 show the calling process when such an attack is successful.

**Figure 6:**
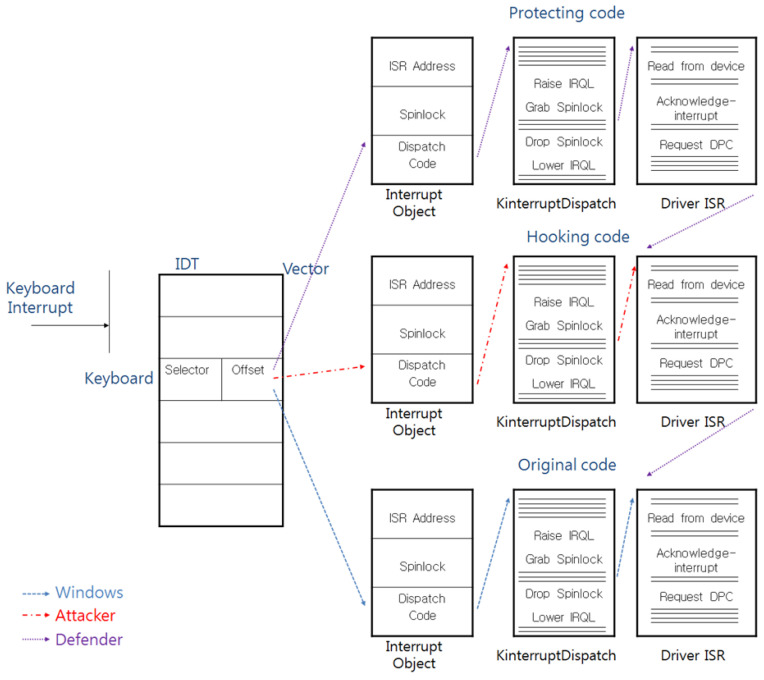
The countermeasure that protects against the IDT-hooking attack.

Attackers can steal keyboard data simply by calling their hooking code before the original code is called; however, a defender can also use this attack technique to protect the keyboard data by calling the protection code before the hooking code is called. Figure 6 shows the process of the protection-code protection technique.

**Figure 7:**
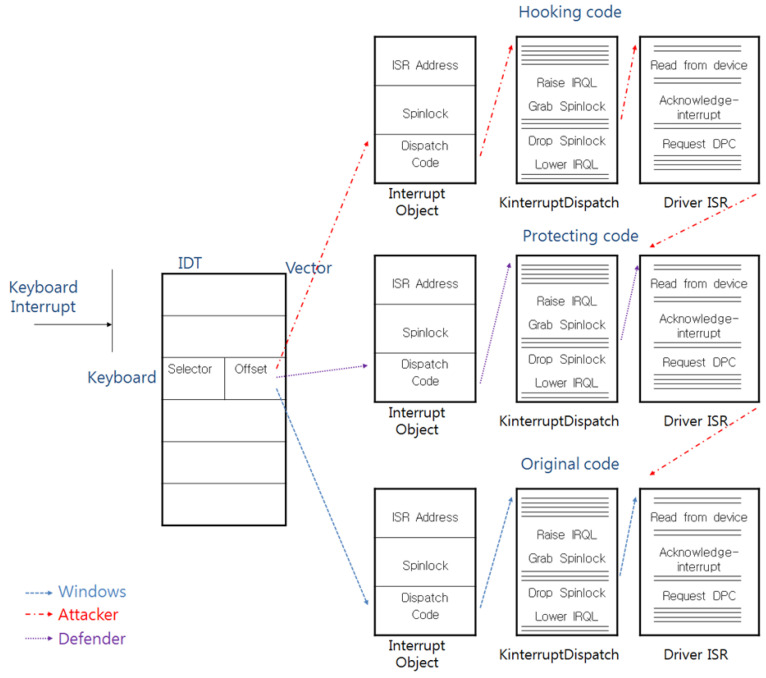
The attack technique that neutralizes the countermeasure against the IDT-hooking attack.

In the execution flow that is shown in Figure 6, a defender protects the keyboard data; however, when the hooking code is executed before the calling of the protection code, the defender cannot protect the keyboard data, and this kind of attack flow is shown in Figure 7.

### 2.2. Direct Polling

Direct polling is a hardware-based attack technique rather than an O/S-based attack technique. For the input and output operations, an atomic operation is specifically prepared in Microsoft Windows, so an attacker can steal keyboard data by periodically reading the keyboard-input data. This attack technique can cause critical results because the O/S also executes the atomic operation for the input or the output so that a defender cannot detect and protect this kind of operation. When direct polling is attempted, the attacker always monitors the state of the keyboard controller, and Table 1 and Table 2 show the keyboard-register information [5,6,11,23].

**Figure 8:**
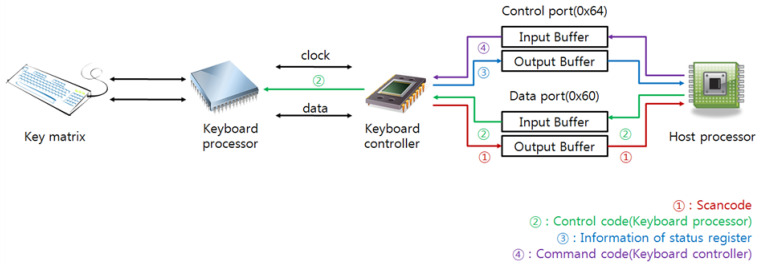
The connection flow between the keyboard and the host.

A host platform prepares the keyboard controller (8259A) inside the host to enable communication between the keyboard and the host, and the control data or the keyboard data are transferred through specific ports (control port: 0x64; data port: 0x60). Each port comprises output and input buffers for the writing or reading of the control data. If the host reads the control port (0x64) or the data port (0x60), the host obtains the status register from either the control port or the data-port scan code. Additionally, if the host writes the control code or a command code to the control port or the data port, it transfers the code to the keyboard controller for the control code or to the keyboard for the command code. The first and second bits of the keyboard-status register represent the OBF and the IBF, respectively. When the OBF or the IBF is set, the output or input buffer is filled with either control or data information; therefore, the attacker can decide whether the keyboard is pressed or not by checking the OBF. If the OBF is set, the attacker can steal the input keyboard scan code by reading the data port. Figure 8 shows the connection flow between the keyboard and the host.

### 2.3. C/D-Bit Vulnerability Utilization

The creation of new defense techniques that can provide protection against the direct-polling attack, whereby the defender confuses the attacker with mingling-noise scan codes, remains the research focus of many security experts. For this technique, the keyboard controller generates random scan codes by using the 0xD2 command code. Table 3 shows the primary command codes of the keyboard controller.

**Figure 9:**
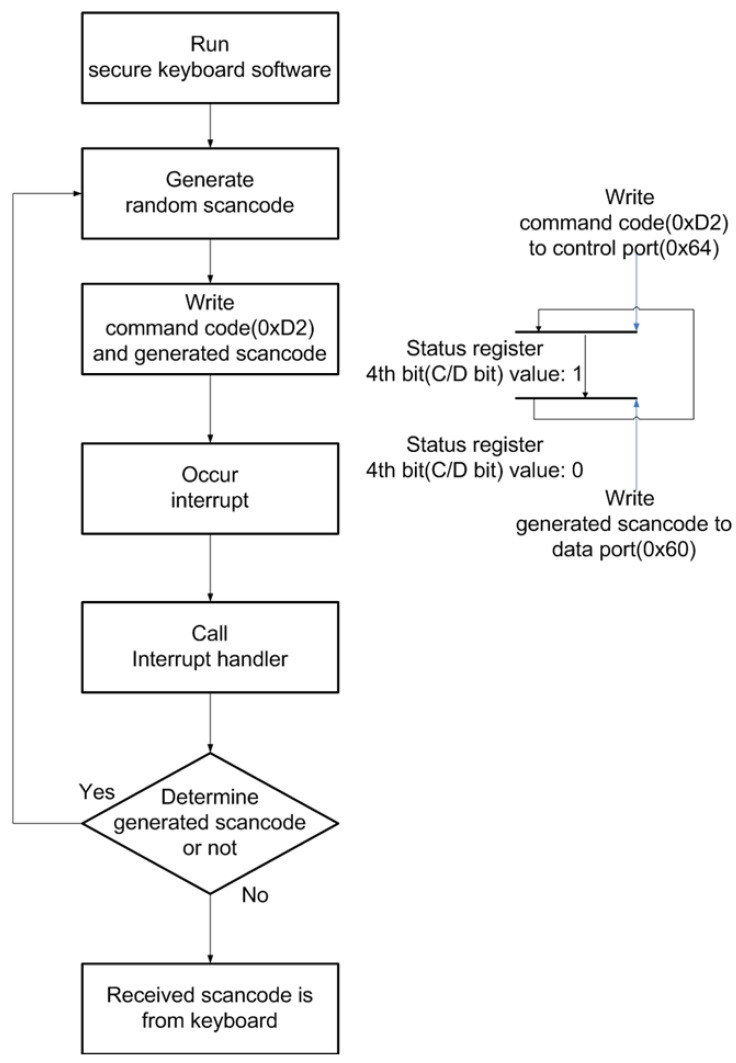
The process of the countermeasure for which mingling-noise scan codes are applied.

The secure-keyboard software generates a random scan code and then writes the 0xD2 command code to the control port; thereafter, the program writes generated scan codes to the data port. As a result, the keyboard controller receives the scan code and then makes a keyboard interrupt. The generated keyboard interrupt calls the keyboard interrupt handler and sends the received scan code to the secure keyboard software. The secure keyboard software confirms whether or not the received scan code is identical to the generated scan code, and if the received scan code is the same, the attacker decides that the user did not press the keyboard. If, however, the received scan code is not the generated scan code, the received scan code is from the user input, and the attacker becomes confused, because if the attacker obtains the scan code by replacing the interrupt object or through direct polling, he or she does not check whether it comes from the keyboard or if it is generated by the secure-keyboard software. This technique is therefore an efficient countermeasure that is very safe. Figure 9 shows the process of the countermeasure for which mingling-noise scan codes are applied.

**Figure 10:**
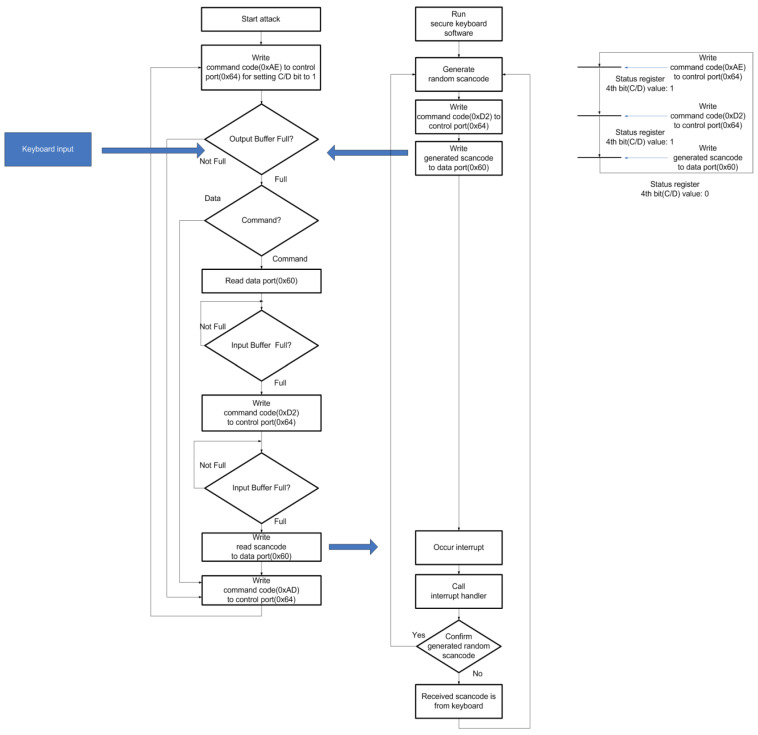
The attack technique using C/D bit vulnerability.

Nevertheless, this solution has vulnerabilities. Keyboard controller has allocated C/D bit (bit 3) in the keyboard status register for decision a received command or a control code comes from. If the keyboard controller receives a specific code through the control port (0x64), C/D bit is set to TRUE. Otherwise, if the keyboard controller receives the specific code through the data port (0x60), C/D bit is set to FALSE. Whereas when secure keyboard software makes mingling noise scan code, the C/D bit makes the polling edge. Therefore, an attacker is able to separate a generated scan codes from a keyboard scan code by ascertaining that whether C/D bit is set TRUE or FALSE. If C/D bit set TRUE, it means that the scan code comes from the keyboard. Otherwise, if C/D bit is set FALSE, it means that the scan code comes from the secure keyboard software. Figure 10 shows the flow of this kind of attack.

### 2.4. Debug Exception Handler Replacement

**Figure 11:**
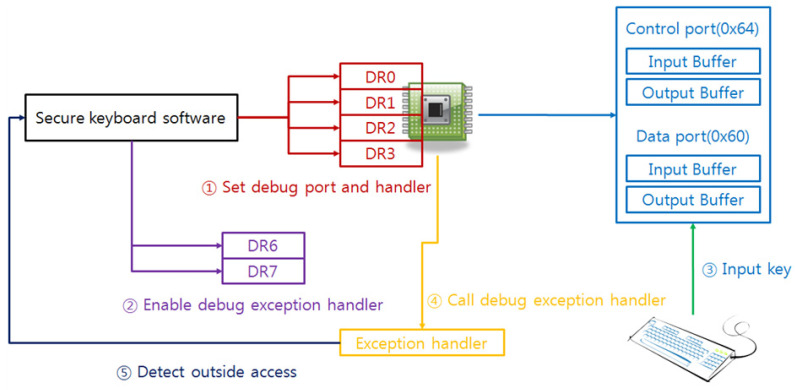
The countermeasure is using the exception handler of debug registers.

C/D bit vulnerability can neutralize generated random scan code without changing C/D bit. In order to solve this problem, defenders can store random keyboard data using writing command code 0x60–0x7F, and then they are able to obtain the written scan code using reading command code 0x20–0x3F because a keyboard processor has internal memory. These command codes transfer to the keyboard directly through the data port (0x60), and command codes and parameters also send though the data port (0x60). This solution generates noise scan codes efficiently because these command codes do not make polling edge. Another countermeasure is utilizing by replacing debug exception handler. Widely used Intel processor in current platform has debug registers for debugging and if the user sets the specific I/O port or the memory to the debug register. After that, someone accesses set the I/O port or the memory, Intel processor occurs an exception and calls set the exception handler. Therefore, the secure keyboard software can protect the keyboard data by setting debug port to 0x60 and 0x64 because an attacker has to access these ports to steal the keyboard data. If the attacker accesses these ports, the exception handler always calls at first, so the secure keyboard software is able to preempt the scan code than the attacker. Figure 11 shows the step of this countermeasure.

**Figure 12:**
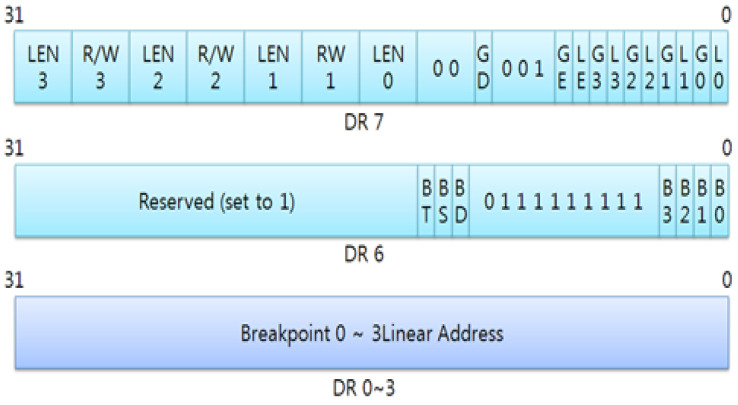
The structure of debug registers.

However, this countermeasure is also able to be used by an attacker, if the attacker uses this protection technique, the secure keyboard software and the attack program have race condition to set debug registers. Especially, the secure keyboard software always executes and terminates specific time, so the attack program can steal scan codes by monitoring input and output ports. Because the attacker can check whether the secure keyboard software runs or not, and if the secure keyboard software is executed after setting debug handler, the attacker is able to set the debug handler. It means that if someone accesses 0x60 and 0x64 ports, Intel processor always calls set the exception handler registered by the attacker. In addition, DR6 and DR7 registers are in charge of activation and deactivation of the exception handler, so the attacker can neutralize the debug handler. Figure 12 shows a structure of debug registers.

### 2.5. Resend Control Code Utilization

A countermeasure using debug exception handler also has vulnerability, but the most serious problem comes from a specific control code. As mentioned in Section 2.3, control codes can send to a keyboard. Table 4 shows this kind of control codes.

**Figure 13:**
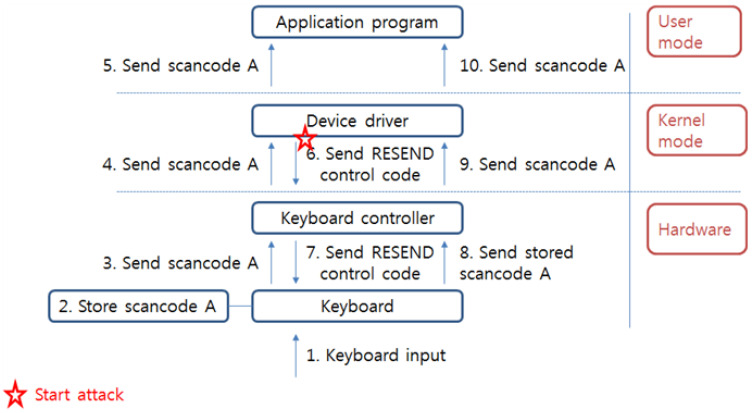
Attack process whereby the RESEND control code is utilized.

There are commands for setting a transmission period when the user presses the key over specific time and other command to set of scan code set. Especially, RESEND control code is a function for resending last sent data when user wants to the keyboard self-test or when the transferred data has error. Through this control code, a keyboard controller restores an error or an incorrect transmitted data or corrupted information by noise. At first, when the keyboard controller is designed, RESEND control code is properly utilized on the positive point of view but if an attacker uses the code for malicious purpose, this code will be a critical problem as vulnerability. When the attacker stole the keyboard scan code by attack techniques such as interrupt object replacement, direct polling, C/D bit vulnerability utilization and debug exception handler replacement at specific time, the attacker can extort really inputted data by comparing obtained scan code with newly received scan code using RESEND control code. This kind of control code sends to the keyboard directly, so a defender is hard to protect this attack because the attack is implemented in a legal way. Therefore, this vulnerability exists continuously, unless keyboard controllers such as 8042 controller and 8259A controller are not changed. Figure 13 shows an attack process by utilizing RESEND control code.

## 3. Security Assessment of the Secure Keyboard Software

This section draws the security assessment based on keyboard attack techniques described above Section 2. We verify exposure of keyboard scan codes by a sample attack program on the running secure keyboard software. In order to experiment the security assessment, we use a laptop installed Windows 7 Home Premium K Service Pack 1 operating system, and this laptop has Intel(R) Atom(TM) 1.60 GHz CPU and 1 GB RAM. The sample attack code is implemented by C and C++ languages, and a compile tool is Visual Studio 2005 for the application program and Windows DDK 3790.1830 for device driver. Experimental subjects are six different famous websites of securities companies and websites of online banking in Republic of Korea, and Paypal, Amazon and other banking sites such as Chase and Purdue federal are included. Table 5 shows experimental results. Detection is that the secure keyboard software detects whether the attacker program is running or not, and exposure is that the attacker program steals the keyboard data even though secure keyboard software is running.

Above experimental results, three secure keyboard programs among all secure keyboard programs did not expose the keyboard scan code, and other three secure keyboard programs did not detect and expose the scan code. This result means that dozens or hundreds of websites are exposed the keyboard scan code inputted from the user because these websites is applied above companies’ programs in Republic of Korea. In addition, international websites do not have any kind of secure keyboard software, and both two online banking sites and two bank sites are exposed the keyboard scan code. Therefore, the Republic of Korea and USA need the countermeasure to protect the keyboard scan code on all websites for providing the internet banking service. Lastly, in terms of detection of the attack program, the problem is that it is not attack techniques. In this paper, we drew experimental results of the detection, or a cleaning of the sample attack programs, as a result, eight anti-virus programs did not detect. Table 6 shows these results. These anti-virus programs focused on a network security, so if attackers store the stolen keyboard data, and then they monitor termination time of the anti-virus software, attackers can send the stolen scan code to themselves without any restriction because sending time is after terminated anti-virus software. The keyboard scan codes are exceedingly related to privacy and financial information such as ID, password, credit card number, credit card CVS, credit card password and so on. Hence, when this kind of data is stolen by a third-party, it causes exceedingly critical damages. In this paper, we do not analyze the reason why scan code is not exposed in some secure keyboard programs. Nevertheless, similar attacks are likely to happen, so we will study this possibility in the future. The fundamental reason for these vulnerabilities is that the hardware chip related to the PS/2 interface keyboard does not provide security facilities. Therefore, since keyboard data exposure does not be prevented only by software, it is required to develop a hardware chip that provides security facilities.

## 4. Conclusions

In this paper, we analyzed keyboard attack techniques and surveyed the current state of secure keyboard programs. Surveyed secure keyboard programs assure the security by certificate in Republic of Korea but recently new keyboard attack techniques have arisen, so a new security assessment is needed with existing attack techniques. At present, a user uses a laptop than a desktop, so this paper is focused on the PS/2 keyboard.

In order to analyze the PS/2 keyboard security, we classified attack and defense techniques according to 10 categories, and we proved the keyboard security based on most utilizing existing attack techniques such as interrupt object replacement, direct polling and new discovered attack techniques such as C/D bit vulnerability utilization, debug exception handler replacement and RESEND command utilization. For this experiment, we investigated and analyzed these attack techniques and implemented sample attack programs with the device driver. After that, we confirmed exposure of the keyboard scan code inputted from the user on online banking websites or banking websites which were running the secure keyboard software. Experimental results show 70% successful with attacks, and in case of secure keyboard programs, these programs are applied to many websites, so this result means that these kinds of attack techniques are an attack level with serious damages. Moreover, anti-virus programs do not detect sample attack programs, so a new countermeasure for this problem is needed quickly.

## Figures and Tables

**Table 1:** The keyboard-register information.

Register	Feature	Description
Status register	Read	Decode-status information
Control register	Read/Write	Store-control information

**Table 2:** Keyboard-status register configuration.

Bit	Field
Bit 0	OBF (Output Buffer Full)
Bit 1	IBF (Input Buffer Full)
Bit 2	System Flag
Bit 3	C/D (Control/Data) bit
Bit 4	Inhibit Switch
Bit 5	Transmit Time-out
Bit 6	Receive Time-out
Bit 7	Parity Error

**Table 3:** Primary command codes of the keyboard controller.

Code	Feature	Parameter	Response
0x20	Read Configuration Register	X	Configuration Register Value
0x60	Write Configuration Register	O	-
0xAA	Self-test	X	0x55
0xAB	Interface Test	X	0x00
0xAD	Disable Keyboard Interface	X	-
0xAE	Enable Keyboard Interface	X	-
0xC0	Read Input Port	X	Input Port Value
0xD0	Read Output Port	X	Output Port Value
0xD1	Write Output Port	O	-
0xD2	Write Keyboard Output Buffer	O	Transferred Parameter

**Table 4:** Primary control codes of keyboard controller.

Code	Feature	Parameter	Response
0xF5	Disable	X	-
0xEE	Echo	X	0xEE
0xF4	Enable	X	0xFA (ACK)
0xF7-0xFD	Set Key Type Make	X	0xFA (ACK)
0xEF-0xF2	Read ID	X	0xAB, 0x83
0xFF	Reset	X	0xFA (ACK)
0xF6	Set Default	X	-
0xF3	Set Typematic Rate/Delay	O	-
0xFA	Set All Keys Typemetic/Make/Break	X	0xFA (ACK)
0xAA	AT Completion Code	X	-
0xF0	Set Scan Code	O	0xFA (ACK)
0xFE	Resend	X	Last Sent Value (Scan code, etc)

**Table 5:** Scan code exposure and detection of the sample attack program (Detection/Exposure).

Company/Attack Technique		Interrupt Object Replacement	Direct Polling	C/D Bit Vulnerability Utilization	Debug Exception Handler Replacement	RESEND Command Utilization
Republic of Korea	Company A	X/X	X/X	X/O	X/O	X/O
	Company B	X/X	X/X	X/X	X/X	X/X
	Company C	X/X	O/X	O/X	X/O	X/O
	Company D	X/X	X/X	X/X	X/X	X/X
	Company E	O/X	O/X	O/X	O/X	O/X
	Company F	X/X	X/X	X/X	X/X	X/O
International	Company G	X/O	X/O	X/O	X/O	X/O
	Company H	X/O	X/O	X/O	X/O	X/O
	Company I	X/O	X/O	X/O	X/O	X/O
	Company J	X/O	X/O	X/O	X/O	X/O

**Table 6:** Detection results of anti-virus software.

Company/Attack Technique	Interrupt Object Replacement	Direct Polling	C/D Bit Vulnerability Utilization	Debug Exception Handler Replacement	RESEND Command Utilization
Company A	X/X	X/X	X/O	X/O	X/O
Company B	X/X	X/X	X/X	X/X	X/X
Company C	X/X	O/X	O/X	X/O	X/O
Company D	X/X	X/X	X/X	X/X	X/X
Company E	O/X	O/X	O/X	O/X	O/X
Company F	X/X	X/X	X/X	X/X	X/O
Company G	X/O	X/O	X/O	X/O	X/O
Company H	X/O	X/O	X/O	X/O	X/O
Company I	X/O	X/O	X/O	X/O	X/O
Company J	X/O	X/O	X/O	X/O	X/O

## Data Availability

The data are contained within the article.
